# Complete abrogation of sporozoite-induced sterile immunity by blood stage parasites of homologous and heterologous malaria species

**DOI:** 10.1186/1475-2875-9-S2-O19

**Published:** 2010-10-20

**Authors:** Megumi Inoue, Jianxia Tang, Osamu Kaneko, Katsuyuki Yui, Richard Culleton

**Affiliations:** 1 Department of Protozoology, Institute of Tropical Medicine, Nagasaki University, 1-12-4 Sakamoto Nagasaki 852-8523, Japan; 2 Department of Molecular Microbiology and Immunology, Graduate School of Biomedical Sciences, Nagasaki University, 1-12-4 Sakamoto Nagasaki 852-8523, Japan

## 

Immunisation of mice and humans with attenuated sporozoites has been shown to confer sterile immunity against infection. There are ongoing efforts to develop a vaccine based on this system. Attenuation of sporozoites may be achieved via irradiation, genetic modification, or through the use of drugs targeting the blood stage parasite. Recently, it has been shown that the administration of chloroquine, a drug that acts exclusively against the erythrocytic stages of malaria parasites, concurrently with live sporozoites can induce sterile immunity against homologous challenge with *Plasmodium falciparum* sporozoites in humans. However, it is not known whether the protection achieved against *P. falciparum* will also protect against other species of human malaria parasites, which are almost always endemic in the same region. Given the high amount of antigen diversity within malaria parasite species, coupled with the large evolutionary distances between the human species, it seems unlikely that immunity to heterologous species would be achieved. Of concern is whether the development of an acute blood stage infection of a heterologous species may abrogate the immunity achieved against the vaccine target species. This phenomenon would have serious consequences for the deployment of an attenuated sporozoite vaccine in a multispecies endemic area.

Here, we describe the results of experiments aimed at determining whether immunity achieved against one species of malaria parasite by sporozoite immunisation is protective against a secondary species, and whether the development of acute blood stage infections of the heterologous species abrogates the immunity achieved against the vaccine target species. As such experiments are currently impossible using human malaria parasite species, we have used the rodent malaria parasite species *Plasmodium vinckei* and *Plasmodium yoelii.* These two species were selected as they offer the widest possible evolutionary distance between rodent malaria parasites, although they are still much more closely related than any two of the human species. We found that although there was some cross protection between the species following sporozoite immunisation in conjunction with mefloquine treatment, the major component of this immunity was species specific. Mice immunised with *P. yoelii* sporozoites were completely protected against the development of blood stage infection following *P. yoelii* sporozoite challenge, whereas they were completely susceptible to infection with *P. vinckei.* Crucially, we found that the development of a blood stage infection of either species completely removed the sterile protection achieved *via* sporozoite immunisation.

